# Intraoperative stapled anastomosis following small bowel perforation: a clinical image

**DOI:** 10.11604/pamj.2026.53.62.49784

**Published:** 2026-02-06

**Authors:** Sachin Bharshankar, Ruchira Ankar

**Affiliations:** 1 Department of Medical Surgical Nursing, Smt. Radhikabai Meghe Memorial College of Nursing, Datta Meghe Institute of Higher Education and Research, Wardha, Maharashtra, India

**Keywords:** Exploratory laparotomy, abdominal closure, linear gastrointestinal stapler

## Image in medicine

A 50-year-old male patient presented with acute onset abdominal pain associated with vomiting, progressive abdominal distension, and failure to pass flatus and feces; and peritonitis due to distal ileal perforation. Emergency exploratory laparotomy with bowel resection and stapled side-to-side anastomosis was performed, followed by thorough peritoneal lavage. There was a history of a long-standing abdominal wall swelling that became painful and irreducible. On examination, the patient was toxic with signs of intestinal obstruction and localized peritonism. Routine demographic identifiers are withheld to maintain anonymity. An emergency exploratory laparotomy was undertaken following clinical and radiological suspicion of acute intra-abdominal pathology. Intraoperatively, a diseased segment of small bowel. Hemodynamic parameters stabilized within the first 24 hours, and a gradual resolution of abdominal pain and distension was the short-term outcome. Among the medium-term outcomes were: successful tolerance of oral feeding by day 4-5; normal bowel movements restoration; complete wound healing without dehiscence; and no anastomotic leak oriented tra-abdominal abscess. The diagnostic approach included: acute abdomen with features of bowel obstruction and strangulation; laboratory investigations: leukocytosis and metabolic derangement suggestive of sepsis; and radiological assessment: abdominal imaging suggestive of obstructed hernia.

**Figure 1:**
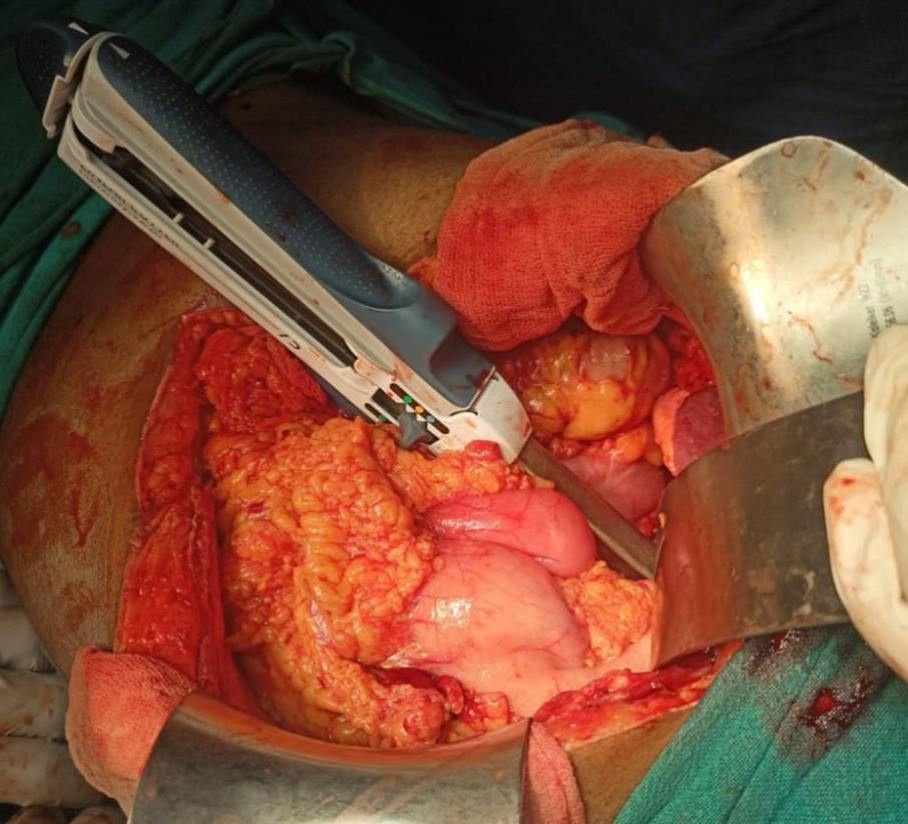
intraoperative view showing stapled side-to-side small bowel anastomosis after resection of the perforated ileal segment

